# Transcriptional orchestration of mitochondrial homeostasis in a cellular model of PGC-1-related coactivator-dependent thyroid tumor

**DOI:** 10.18632/oncotarget.24633

**Published:** 2018-03-23

**Authors:** Solenne Dumont, Soazig Le Pennec, Audrey Donnart, Raluca Teusan, Marja Steenman, Catherine Chevalier, Rémi Houlgatte, Frédérique Savagner

**Affiliations:** ^1^ L’institut du Thorax, INSERM, CNRS, UNIV Nantes, BP 70721, 44007 NANTES Cedex 1, France; ^2^ Inserm UMR 1048, I2MC, 31432 TOULOUSE Cedex 4, France; ^3^ UMS 016, SFR Santé, IRS UN, BP 70721, 44007 NANTES Cedex 1, France; ^4^ Inserm UMR 954, Faculté de Médecine, BP 184, 54505 VANDOEUVRE-LÈS-NANCY Cedex, France

**Keywords:** PGC-1-related coactivator, transcription factors, miRNAs, integrative network

## Abstract

The PGC-1 (Peroxisome proliferator-activated receptor Gamma Coactivator-1) family of coactivators (PGC-1α, PGC-1β, and PRC) plays a central role in the transcriptional control of mitochondrial biogenesis and oxidative phosphorylation (OXPHOS) processes. These coactivators integrate mitochondrial energy production into cell metabolism using complementary pathways. The XTC.UC1 cell line is a mitochondria-rich model of thyroid tumors whose biogenesis is almost exclusively dependent on PRC. Here we aim to propose an integrative view of the cellular pathways regulated by PRC through integration of cDNA and miRNA microarray data and chromatin immunoprecipitation results obtained from XTC.UC1 cells invalidated for PRC. This study showes that PRC induces a complex network of cellular functions interacting with at least one to five of the studied transcription factors (Estrogen Related Receptor alpha, ERR1; Nuclear-Respiratory Factors, NRF1 and NRF2; cAMP Response Element Binding, CREB; and Ying Yang, YY1). Our data confirm that ERR1 is a key partner of PRC in the regulation of mitochondrial functions and suggest a potential role of this complex in RNA processing. PRC is also involved in transcriptional regulatory complexes targeting 12 miRNAs, five of which are involved in the control of the OXPHOS process. Our findings demonstrate that the PRC coactivator can act in complex with several transcription factors and regulate miRNA expression to control the fine regulation of main metabolic functions in the cell. Therefore, in PGC-1α/β-associated pathologies, PRC, as a metabolic sensor, may ensure mitochondrial homeostasis.

## INTRODUCTION

Metabolic adaptation involves regulation of energy homeostasis at the transcriptional and postranscriptional levels using diverse transcription factors, coregulators and feed-back control loops [1]. Mitochondrial oxidative phosphorylation (OXPHOS) is central for energy homeostasis and is the only process under dual genetic control in mammals: thirteen essential structural subunits are encoded by mitochondrial DNA while remaining subunits are nuclear-encoded and imported into the mitochondria. The mechanism controlling nucleus-mitochondrial crosstalk may need co-expression of many factors considering a long-term adaptive response for mitochondrial biogenesis. Studies on several transcription factors regulating mitochondrial biogenesis and function have shown extensive use of feed-forward and feed-back loops to control this biogenesis [2–4]. It has been shown that Nuclear-Respiratory Factors (NRF1 and NRF2/GABP), Estrogen Related Receptor alpha (ERR1), Ying Yang (YY1) and cAMP Response Element Binding (CREB) ensure effective coordination of nuclear and mitochondrial gene expression [5]. Master transcriptional coregulators like Peroxisome proliferator-activated receptor Gamma Coactivator 1 (PGC-1α) and related family members (PGC-1β and PGC-1-Related Coactivator or PRC) may interact with some of these transcription factors and exhibit a unique capacity to control complex transcriptional networks and remodel the metabolic landscape.

The functional properties of mitochondria in cells preferentially expressing either PGC-1α, or PGC-1β or PRC differ in terms of OXPHOS efficiency and oxidative stress defenses, suggesting that these coactivators induce similar but not identical programs [6, 7]. Knockdown studies have demonstrated that the three coactivators are able to compensate each other [8–10]. It has been suggested that if PGC-1α and PGC-1β are essential to drive mitochondrial biogenesis in tissues with high-energy demand, another member of the family ensures the basal level of mitochondrial biogenesis. Previous results have underlined the fine regulation of mitochondrial biogenesis and function exerted by the ubiquitous and serum-dependent PRC coactivator when PGC-1α and PGC-1β were underexpressed using animal and cellular models [7, 11]. However, few studies have explored the combined effect of multiple regulators on mitochondrial biogenesis and function [8, 12, 13], revealing unexpected roles of the combination of factors in energy homeostasis.

We have previously identified a cellular model of thyroid tumor characterized by a high content of functional mitochondria with almost exclusive PRC-dependent biogenesis [7]. Microarray and mitochondrial function analyses exploring both mitochondrial ATP synthesis and oxidative phosphorylation processes of this XTC.UC1 cell line have suggested a temporal regulation of the PRC coactivator through several transcription factors. Here we used an integrative genomic approach combining Chromatin ImmunoPrecipitation (ChIP)-on-chip and cDNA and miRNA microarray tools to explore transcriptional and post-transcriptional regulations of the PRC-dependent network.

## RESULTS

### ChIP-chip analyses in XTC.UC1 cells

**Figure 1:**
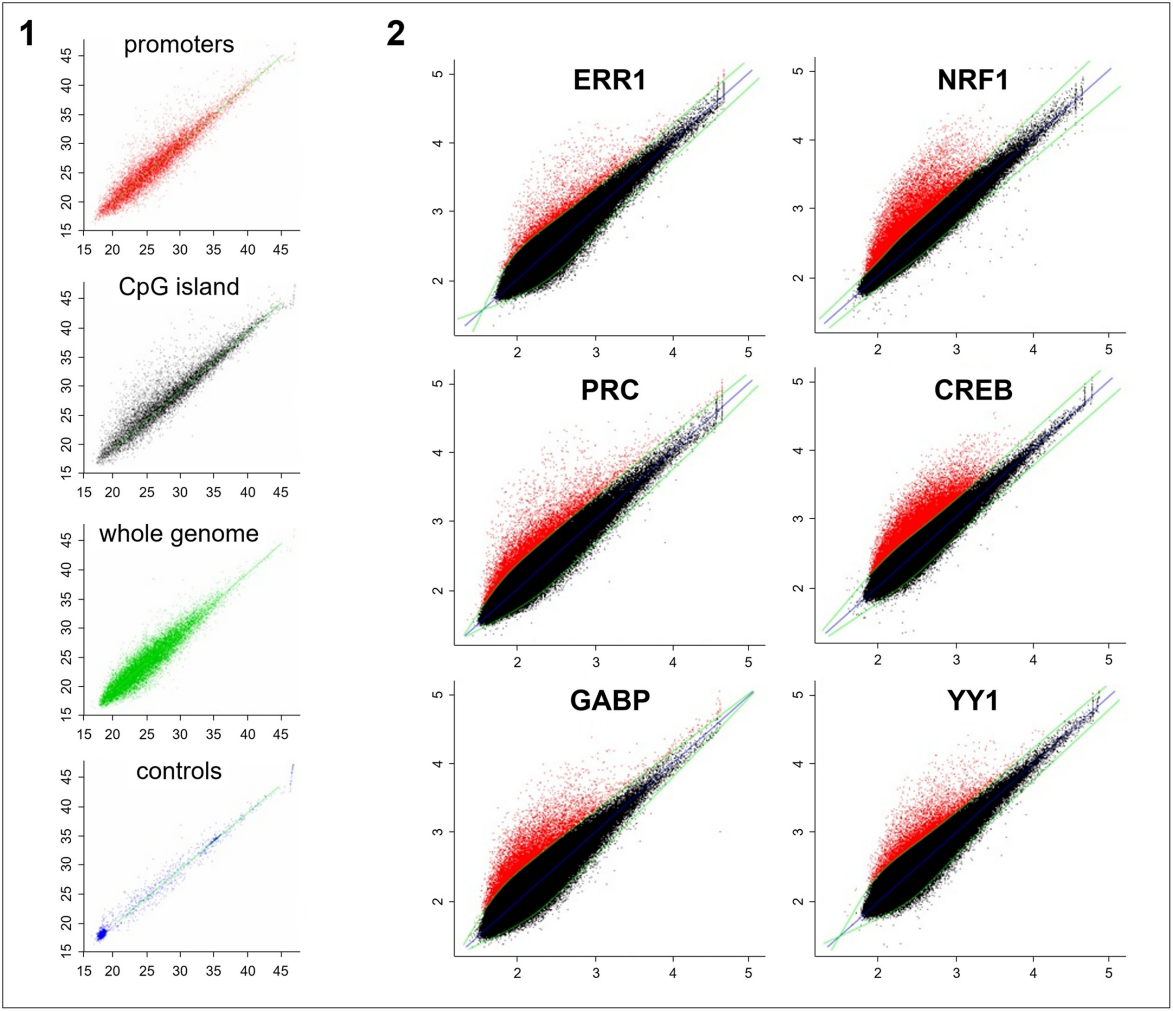
ChIP-chip analysis of the five transcription factors (NRF1, GABP/NRF2, ERR1, CREB and YY1) and the PRC coactivator in XTC.UC1 cells after 48h of serum induction Immunoprecipitated fractions of XTC.UC1 cells using PRC, ERR1, NRF1, CREB, GABP or YY1 antibodies were hybridized on custom hybrid chips (1, results shown only for GABP) and promoter arrays (2). Horizontal axis corresponds to probe signal intensities from the whole chromatin fraction and vertical axis to those of the immunoprecipitated fraction. 1-1: Custom hybrid chips consisted of CpG islands (10,000, black points), specific gene promoters (10,000, red points), random sequences (20,000, green points) and several negative controls (blue points) relative to repeated non-transcribed regions of the genome. Polynomial regression curve (green) led to the selection of positive probes from background by using a GABP antibody for chromatin immunoprecipitation. 1-2: Promoter arrays consisted of 244,000 probes corresponding to nearly 17,000 gene promoters (Agilent Human Promoter ChIP on chip arrays 244K). The blue straight line represents identity, while the green curve is a spline fit through the scatter plot separating background (black) from positive probes (red) for each antibody used. Positive probes were selected using statistical tests adapted to non-tiling chips.

**Figure 2:**
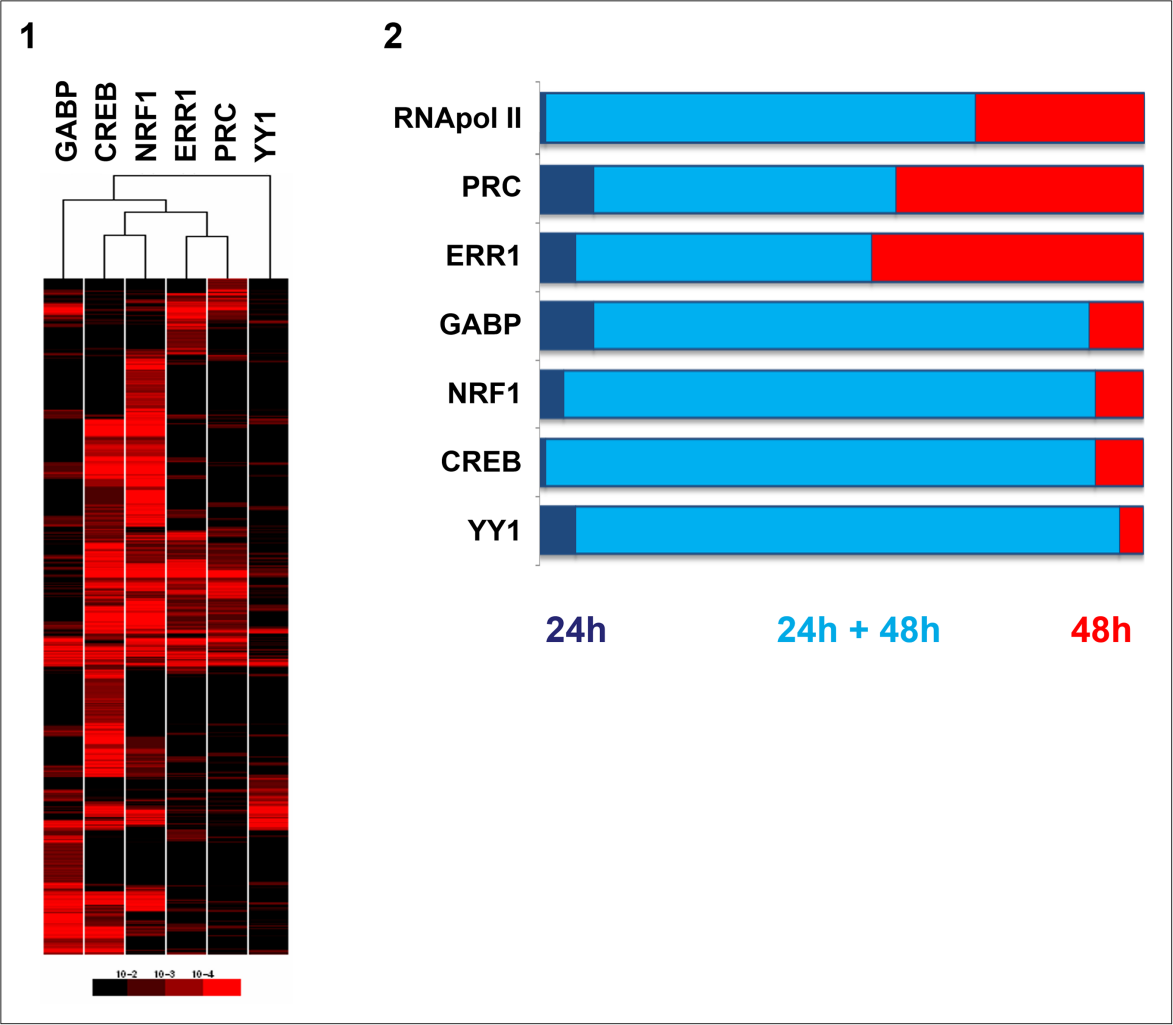
Comparison of gene-regulation of the five transcription factors (GABP, CREB, NRF1, ERR1, YY1) and the PRC coactivator in XTC.UC1 cells after serum induction through ChIP-chip analysis 2-1: Hierarchical clustering of the p-value matrix for genes positive on ChIP-chip after 48h of serum induction for the six factors. The p-value of geometric means for the best probe of two technical replicates was transformed in red color intensities and clustered. Each line corresponded to the p-value of a positive gene for at least one factor. Columns represent factors. 2-2: Stacked bar chart representing the proportion of genes positive on ChIP-chip at 24h and/or 48h of serum induction. ChIP-chip for RNA polymerase II (RNApol II) was used as a positive control for active gene transcription. Proportions of genes positive on ChIP-chip only at 24h of serum induction (dark blue), those positive both at 24h and 48h of serum induction (light blue) and those exclusive to 48h of serum induction (red) are shown.

Using ChIP-chip, we identified genomic targets of the PRC coactivator and of five transcription factors (CREB, ERR1, GABP, NRF1, YY1) previously predicted to interact with promoters of PRC-regulated genes in XTC.UC1 cells [7]. For each of the factors, the type of genomic regions targeted for transcriptional regulation was first explored using custom chips. We observed that all factors preferentially bound to specific regions (CpG islands and gene promoters) while none interacted with random sequences throughout the whole genome (Figure 1-1). Searching for new target genes regulated by these factors, we then hybridized samples to promoter arrays representing target regions covering 8 Kb around the Transcription Start Site (TSS). For all factors, immunoprecipitation led to an enrichment of target genes when compared to input DNA (IP) (Figure 1-2, red points). Positive probes were selected using a polynomial regression curve and p-values for each probe were calculated relative to the threshold of significance. Geometric means of p-values issued from the two ChIP replicates were considered when p ≤10^−3^. We selected 1,752, 6,707, 3,090 and 2,991 genes positive for the ERR1, NRF1, GABP and YY1 transcription factors respectively. For the CREB factor, known to interfere with several pathways (e.g. regulation of cell cycle), we identified 6,412 positive genes. Finally, 1,951 target genes were selected for the PRC coactivator. Ontology analysis of identified target genes revealed a significant association (p<10^−3^) with oxidative phosphorylation, mitochondrial biogenesis, cell cycle (G1/S and M phases) and MAP Kinase/phosphatase pathways (Supplementary Figure 4). These results validated our strategy for the identification of genes regulated by the five transcription factors and PRC.

### PRC-related transcriptional network

Hierarchical clustering of the p-value matrix corresponding to all positive probes after 48h of serum induction revealed groups of genes co-regulated by several transcription factors (Figure 2-1). This approach also showed that each of the factors could be individually positive for independent genes. Few genes (n=82) were positive only for PRC suggesting that other transcription factors than those we studied interact with this coactivator. The combination of the six factors was associated to promoter regions of 43 genes. One group of genes (n=354) was related to five factors: PRC, ERR1, GABP, CREB and NRF1. Two main groups associated the PRC/ERR1 complex to either CREB/NRF1 (686 genes) or to GABP (246 genes). The Gene Ontology Database was queried using GoMiner software and p-values were computed for each GO term based on the Fisher’s exact test [14]. The main biological processes for different factor combinations were: OXPHOS and mitochondrial biogenesis (p=0.0001) for PRC/ERR1/GABP/CREB/NRF1, M phase and cell communication (p=0.001) for PRC/ERR1/CREB/NRF1 and apoptosis and regulation of programmed cell death (p=0.0003) for PRC/ERR1/GABP. ChIP-chip correlations between genes positive for two transcription factors were tested for all factor combinations (Supplementary Figure 1). A strong correlation was observed for genes regulated by PRC and ERR1. This was also observed when comparing ChIP results for all factors at 24h and 48h of serum induction to ChIP results for RNA polymerase II representing transcriptionally active genes (Figure 2-2). The transcription status was identical after 24h and 48h of serum induction (2-2, clear blue) for the majority of genes regulated by GABP, NRF1, CREB and YY1, whereas PRC and ERR1 also drove transcription of many genes only at 48h (2-2, red). The number of gene promoters positive for each factor at 24h and/or 48h of serum induction is specified in Supplementary Figure 2.

### Promoter analyses

**Figure 3:**
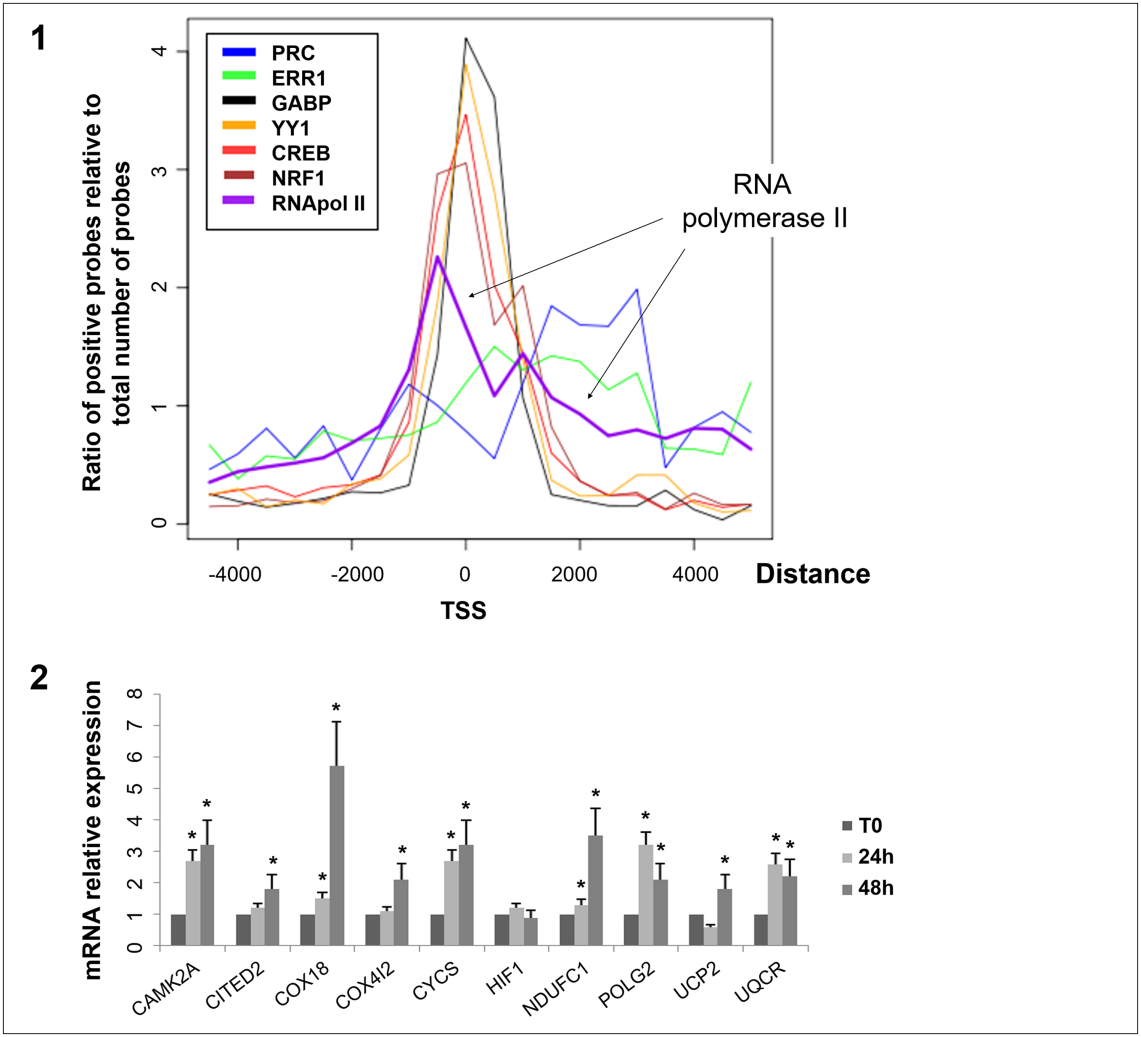
Distribution of distance from the transcription start site for positive probes on ChIP-chip and quantitative RT-PCR results of 10 genes positive for the PRC/ERR1 complex 3-1: Each curve corresponds to the ratio of positive probes on ChIP-chip analysis for each factor relative to the total number of probes at a given distance from the Transcription Start Site (TSS) (log scale). ChIP-chip was performed on XTC.UC1 cells after 48h of serum induction. 3-2: mRNA expression monitored by quantitative RT-PCR. mRNA expression of 10 genes (CAMK2A, CITED2, COX18, COX4I2, CYCS, HIF1, NDUFC1, POLG2, UCP2, UQCR) positive for PRC and ERR1 at 24h and 48h of serum induction relative to T0 (^*^, p≤ 0.05). mRNA expression was relative to beta-globin expression.

Sequence analysis of probes positive in ChIP-chip analysis of transcription factors NRF1, GABP, CREB and YY1 showed that the majority of probes were located in the proximal promoters of genes (Figure 3-1). Using our motif discovery strategy, we confirmed for each factor that its binding site motif was present in the positive probes (Supplementary Figure 3). Interestingly we often identified motifs for other positive studied transcription factors in the vicinity. In contrast, probes positive for ERR1 and PRC were mainly located in the first exon of genes. We also found that the majority of probes positive for RNA polymerase II were located in the proximal promoters of genes (2/3 of genes between −500 and 0 bp from TSS). The remaining RNA polymerase II positive probes displayed a distance profile similar to PRC and ERR1 (0 to 3,000 bp from TSS). This suggests that genes regulated by the PRC/ERR1 complex are associated with RNA polymerase II and are actively transcribed. To confirm the effectiveness of the PRC/ERR1 complex we used quantitative RT-PCR analysis to explore the transcription level of ten genes positive for both PRC and ERR1 on ChIP at 24h and 48h of serum induction. We showed that 6 genes were significantly upregulated at 24h of serum induction and 9 genes at 48h (Figure 3-2).

### Integrative analysis of miRNA expression and ChIP-chip data

**Figure 4:**
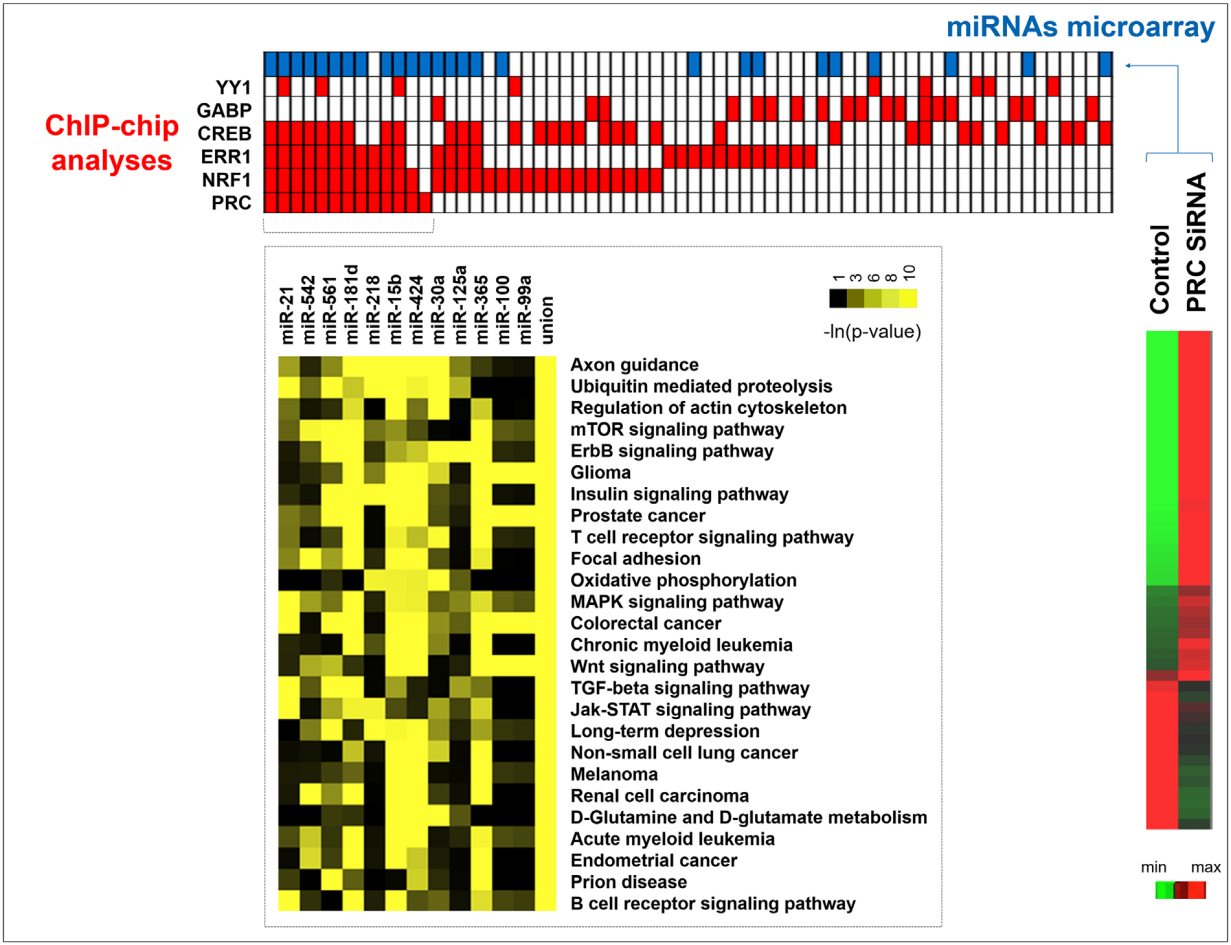
Integrative analysis of miRNA expression and ChIP-chip data MiRNA promoters positive in ChIP-chip analysis after 48h of serum induction in XTC.UC1 cells (red squares) were aligned with the corresponding miRNAs differentially expressed between PRC SiRNA and control XTC.UC1 cells (blue squares) identified by miRNA microarray analysis. For the 12 miRNAs genes identified to be PRC-regulated by the two methods, the Diana miRpath application was used to determine pathway enrichment.

ChIP-chip analyses led us to identify 66 promoters of miRNA genes that were regulated by at least one of the six factors we studied (Figure 4). PRC was associated with the regulation of 13 miRNAs in combination with NRF1, ERR1, CREB or YY1 but not with GABP (Figure 4, red squares). For the regulation of 12 of these miRNAs (miR-21, 542, 561, 218, 15b, 424, 365, 181d, 30a, 125a, 100, 99a) the involvement of PRC was underlined by miRNA microarray analysis at T48 of PRC down-regulation in XTC.UC1 cells (Figure 4, blue squares for miRNAs differentially expressed after down-regulation of PRC). Focusing on these 12 miRNAs, we used the Diana miRpath application to define pathways enriched among the predicted targets [15]. The 101 identified pathways were filtered down to 26 by additionally requiring that putative miRNA targets were enriched in at least 3 of the 101 pathways ((-ln(p-value))>3). Results of the analyses were converted into a heatmap using the -ln(p-value) and were clustered on the putative miRNA targeted pathway axis (Figure 4). Figure 4 shows that oxidative phosphorylation and proliferative signaling pathways were enriched among the targets of the 12 miRNAs. Union was representative of merge miRNAs representation.

### Integrative analysis of mRNA expression and ChIP-chip data

**Figure 5:**
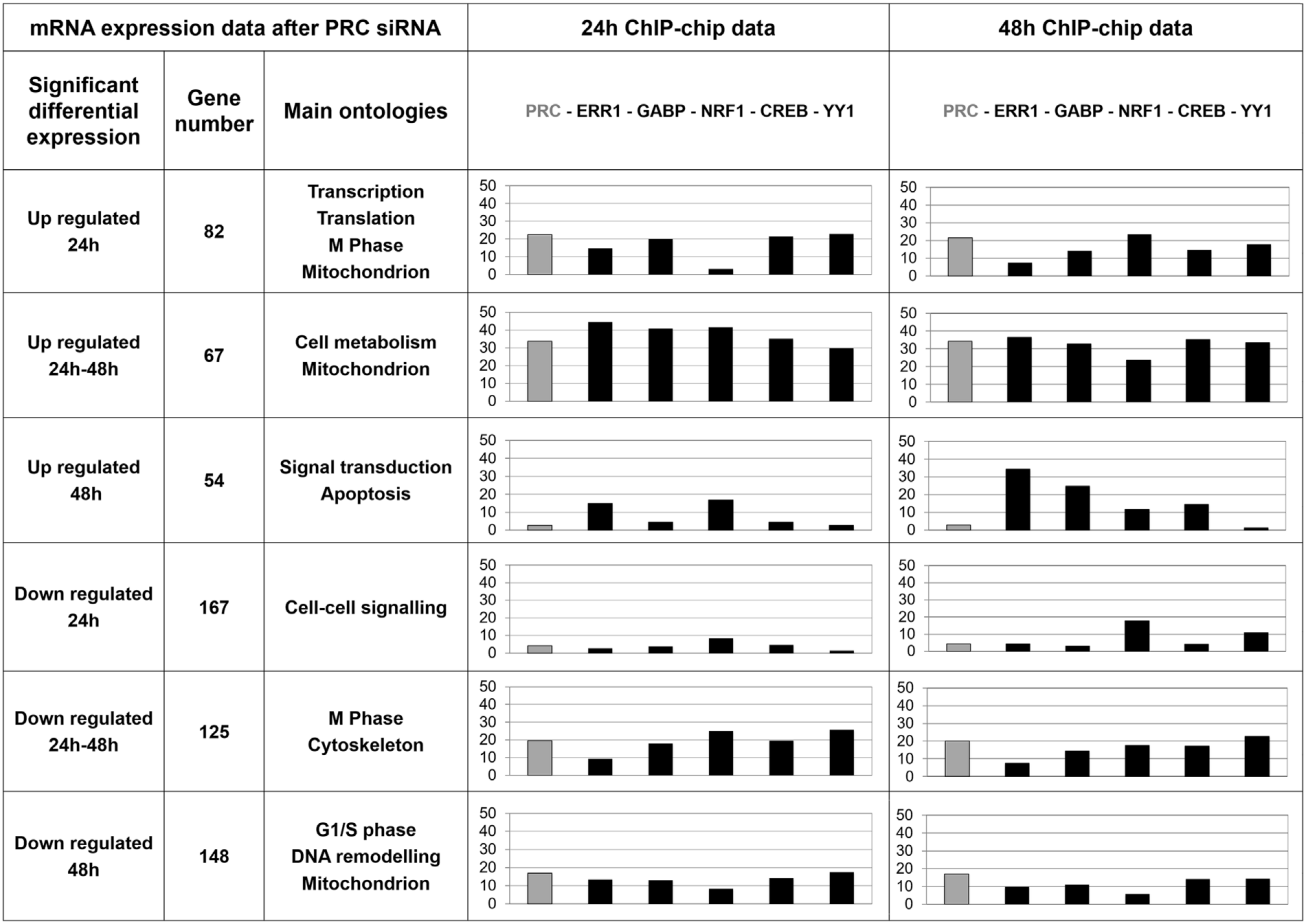
Integrative analysis of transcriptome and ChIP-chip data Main gene ontologies associated to groups of up or down regulated genes in XTC.UC1 cells after PRC invalidation by SiRNA at 24h and/or 48h of serum induction are indicated. In these lists of up and down PRC-regulated genes the percentage of genes positive on ChIP-chip after 24h or 48h of serum induction was determined for the six factors studied (PRC, ERR1, GABP, NRF1, CREB and YY1) and represented by the bar charts.

In order to study ChIP-chip data by a different experimental approach, we combined mRNA expression analysis of temporal inhibition of PRC and ChIP-chip analyses for the six factors (Figure 5). The percentage of genes positive for one or several factors on ChIP-chip and related to ontologies concordant with a previous independent microarray approach [7] underlined the relevance of the antibodies used for ChIP-chip analysis and the strong involvement of factor combinations in cell metabolism regulation. We show that PRC has both positive and negative effects on gene transcription. Six groups of genes with either up or down regulated expression at both T24 and T48 of PRC SiRNA treatment or at only T24 or T48 were identified. Main pathways regulated by PRC were associated with mitochondrial functions and cell cycle checkpoints. Within each of the six gene groups, we determined the percentage of positive genes on ChIP-chip for the six factors studied.

Different profiles of factor combinations were observed. For genes that were up- or down-regulated at both T24 and T48 of PRC inhibition, similar percentages of positive genes on ChIP-chip were observed after 24h and 48h of serum induction. The 5 transcription factors and PRC were present on promoters of 30 to 45% (ChIP 24h) and 24 to 37% (ChIP 48h) of differential genes involved in cell metabolism and mitochondrion pathways. Two groups of genes (up regulated at T24 and down regulated at T48) managed mitochondrial function in the context of cell cycle phases. For genes that were specifically up regulated at T24 of PRC inhibition, a dynamic profile of factor combinations was observed: PRC, ERR1, GABP, CREB and YY1 were present on promoters of 15 to 23% of genes while NRF1 was present in 3% of genes after 24h of serum induction. After 48h of serum induction PRC, GABP, NRF1, CREB and YY1 were present on promoters of 14 to 24% of genes while ERR1 was present in 8% of genes.

## DISCUSSION

We have explored the PRC-related network in a model of thyroid cell tumors with a high content of functional mitochondria. Using both transcriptomic and quantitative PCR analyses we have previously shown that the XTC.UC1 cell line expresses very low levels of PGC-1α and −1β factors compared to the PRC expression level [7, 16, 17]. Thus this cellular model is relevant to study transcriptional and post-transcriptional factors that are able to conduct PRC-dependent mitochondrial biogenesis and function because of its high dependence on this ubiquitous and serum induced member of the PGC-1 family of coactivators [17].

ChIP-chip analyses confirmed that NRF1 and GABP are able to interact with PRC and to regulate several functions related to metabolism and cell proliferation as previously described [8, 11, 18]. We also revealed that YY1 is able to interact with the same DNA regions as PRC as has already been shown for PGC-1α [7]. Clusters of functional transcription factors have been described previously for cooperative activation by ERR1, STAT3 and CREB related to PGC-1α activation [19–21]. Here we showed that cooperative regulation is also relevant for PRC with a complex combination involving up to five transcription factors included in our analysis. PRC and PGC-1α protein sizes are respectively 1,664 and 798 amino acids suggesting that PRC may interact with more transcription factors. However, only 4.2% of the PRC-positive genes (82 out of 1,951) did not require any of the transcription factors we studied for their regulation. Thus our study described for the first time the nearly complete PRC network in the XTC.UC1 model.

Our study showed that ERR1 plays a specific role in PRC-related effects since we frequently identified docking of ERR1 and PRC on the same DNA region. Mechanisms privileging physical interactions between PGC-1 coactivators and ERRs have been previously described with a specific interest for PGC-1α/ERR1 in the control of energy metabolism [22]. Here we showed the existence of an interaction between PRC and ERR1 involved in the control of energy production, cell metabolism and cell proliferation. We have previously shown that the PRC/ERR1 complex is also efficient to mediate the biogenesis of functional mitochondria in cellular models of thyroid tumors [17]. It has been shown that promoter-driven effects on splice site selection occurring through interactions between transcription and splicing machineries are modulated by transcriptional activators such as PGC-1α [23]. This activity has been related to Arg/Ser and Proline-rich regions of the PGC-1α coactivator [4]. Such regions are also present in PRC which contains an extended Proline-rich region that could be related to an RNA processing function of this coactivator and to its location on DNA regions actively transcribed by RNA polymerase II. Persistent co-localization of ERR1 and PRC in those DNA regions could be considered as a stabilizing factor during RNA processing.

Since the PGC-1 family of coactivators plays an integrative role in the cell metabolic network, the direct control of miRNA expression by these coactivators would improve the regulation of the energetic pathways involved in the metabolic switch observed in cancer cells [24]. Depending on short- or long-term regulation, i.e. half time of each of the actors, miRNA-mRNA interactions should be considered for their harmonizing role in a metabolic process, more than for their individual interactions. In thyroid tumors, recent studies have proven the utility of a integrative approach to identify more relevant diagnostic or therapeutic targets [25, 26]. Our study indicates that 12 PRC-regulated miRNAs control cell proliferation as well as OXPHOS at the post-transcriptional level. This suggests that the PRC pathway plays a central role in the integration of mitochondrial biogenesis and energy production and cell proliferation. Five of the 12 miRNAs are specifically involved in the regulation of mitochondrial energy production. Two of these (miR-15b and miR-30a) have been shown to ensure mitochondrial integrity and the regulation of the fusion/fission process [27, 28], while miR-125a belongs to the mitochondrial pool of miRNAs that could directly target some of the mitochondrial DNA-encoded genes of the respiratory chain complexes [29]. Although miR-218 has not been shown to directly target proteins of the mitochondrial respiratory chain, it has been described to target Rictor, the regulatory element of mTORC2, involved in the activation of the mTOR-Akt pathway [30]. Of the two structurally and functionally distinct complexes mTORC1 and mTORC2, the former directly inhibits the effect of PGC-1α-YY1, decreasing the expression of mitochondrial genes [31]. Therefore, the miR-218-mTOR-PRC related pathway must be associated with the regulation of mitochondrial functions.

**Figure 6:**
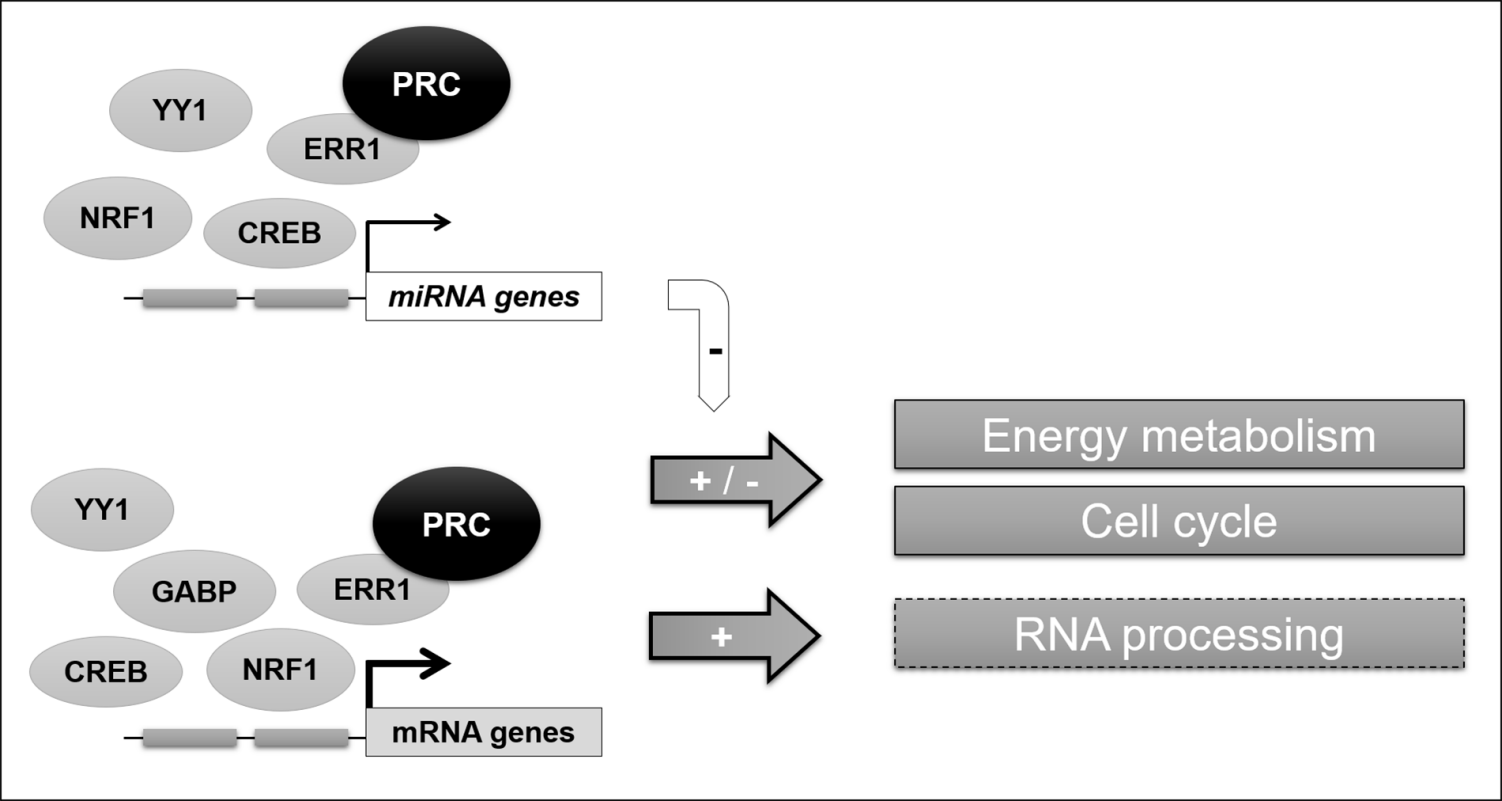
General scheme of the PRC-regulated transcriptional network in XTC.UC1 cells The PRC coactivator can act in complex with several transcription factors, interacting with at least one to five of the studied transcription factors (ERR1, NRF1, GABP, CREB and YY1) to regulate both mRNA and miRNA expression in XTC.UC1 cells. This permits to control the fine regulation of main metabolic functions in the context of cell cycle phases. Our data also confirm that ERR1 is a key partner of PRC for the regulation of mitochondrial functions and suggested a potential role (hatched box) of the PRC-ERR1 complex in RNA processing.

In the present study we have identified complex activities of the ubiquitous member of the PGC-1 family of coactivators, in particular for the control of mitochondrial function through miRNA expression regulation. Our results also underline the relevance of the role of the PRC/ERR1 complex in the control of transcription factor docking and RNA processing for specific functions of PRC in proliferative cells in complement to those of other members of the PGC-1 family. In a general scheme (Figure 6) we conclude on the complex mechanisms for PRC regulation observed in this cellular model.

## MATERIALS AND METHODS

### Cell line and treatments

The human XTC.UC1 cell line was established from an oncocytic cell thyroid carcinoma. The growth medium consisted of Dulbecco’s modified Eagle medium (DMEM) supplemented with 10% foetal calf serum (Seromed, Merck, Darmstadt, Germany), 100 U/mL penicillin, 100 mg/mL streptomycin, 0.25 mg/mL fungizone, and 10 mU/mL thyrotropin (TSH) (Sigma-Aldrich, Saint Louis, MO, USA). Except for the TSH and the foetal calf serum, all the products were obtained from Gibco BRL (Life Technologies, Thermo Fisher Scientific, Waltham, Massachusetts, USA).

To knock down PRC expression in the XTC.UC1 cells, 3 nM of PRC SiRNA (#121729, Life Technologies) was chosen to induce at least 60% of PRC protein inhibition compared to scramble control after 24h of treatment using the protocol previously described [7]. Because of cell cycle-dependent PRC expression, this was a two-step protocol with serum induction followed by SiRNA treatment. We refer to the times of serum induction (T24 and T48) instead of times of SiRNA treatment in the figures and the text.

### PRC SiRNA and cDNA microarray analyses

Total RNA was extracted using Trizol LS reagent (Life Technologies) following the manufacturer’s recommendations. cDNA microarray slides were prepared at the Biogenouest Genomics Core Facility of the University of Nantes (France), using a set of 20,000 oligonucleotides with full functional characterization and content referencing (OciChip microarrays and Ocimum Biosolutions, Hyderabad, India). mRNA amplification from non-transfected cells, PRC SiRNA and scramble-transfected cells, cDNA labelling and hybridization were performed using protocols described by manufacturers (GSE14282 in NCBI’s Gene Expression Omnibus). Bioinformatics analysis has already been described in our previous study [7]. Gene ontology enrichments in gene lists were determined using GOMiner.

### MiRNA microarray profiling

MiRNA was issued from the Trizol-extracted fractions of PRC SiRNA and scramble-transfected XTC.UC1 cells after 48h of 20% serum induction. Microarrays were manufactured by Agilent Technologies (Santa Clara, CA, USA) and contained 20 to 40 features targeting each of 866 human miRNAs. Labeling and hybridization of RNA samples was performed according to the manufacturer’s protocol. Measures were obtained in triplicate. Microarray results were analyzed using GeneSpring GX 7.3.1 software (Agilent Technologies) and are available in NCBI’s Gene Expression Omnibus through GEO Series accession number GSE33843. The Agilent algorithms estimated a single intensity measure for each microRNA, referred to as the total gene signal (TGS) calculated by multiplying the total probe signal by the number of probes per gene. For comparison of hybridizations, the natural logs of the total gene signals for all genes expressing > 10 times the background level were regressed against each other, and the standard deviation of the residuals from the regression line were reported as the root mean square deviation. Clustering was carried out using Cluster 3.0 and visualized using Java Treeview version 1.6 (Eisenlab). The Diana miRpath v2.0 application was used to determine pathway enrichment among the putative targets of miRNAs differentially expressed in PRC SiRNA-treated cells [15].

### ChIP-chip analysis

ChIP assays were performed on 10^6^ XTC.UC1 cells/assay after 24h and 48h of 10% serum induction following the protocol provided by the manufacturer (EZ-ChIP, Upstate, Merck) after crosslink by 1% formaldehyde and sonication. These time-points were selected to ensure complete transcriptional effect of PRC and to address posttranscriptional regulation. Depending on the factor two or three antibodies were tested and antibodies were selected based on two criteria: successful use in previous relevant ChIP studies and/or the relevance of identified functional ontologies or targeted genes. Functional ontologies were either described in the literature or confirmed by our previous PRC-related transcriptomic study [7] using GSEA software to identify over-represented transcription factor binding sites in co-expressed genes [32]. The antibodies were purchased from Santa Cruz Biotechnology (Dallas, Texas, USA), Abcam (Cambridge, UK) and Perseus Proteomics Inc. (Ramona, CA, USA). They were directed against NRF-1 (Ab34682), GABP/NRF-2 (sc-22810), ERR1 (H5844), CREB (ab32515) and YY1 (ab12132) transcription factors and RNA polymerase II (sc-899x). Polyclonal PRC antibody was produced (Eurogentec, Seraing, Belgium) against a human peptide (1520-1534) that we had previously selected. The rabbit anti-goat IgG – used as non-specific immunoprecipitated (IP) control – was purchased from MP Biomedicals (Santa Ana, CA, USA; cat# 55335). All transcription factors were tested in duplicate on two independent XTC.UC1 cell cultures at 70% confluence. For PCR analysis of the ChIP samples prior to amplicon generation, QIAquick-purified IPs were dissolved in 50 μl H_2_O (Qiagen, Hilden, Germany). Quantitative PCR reactions on 6 μl of suitable dilutions from the IP and the input DNA were performed using primers designed for genes known to be targeted by each of the transcription factors studied. ChIP was considered positive after identification of at least a 3-fold enrichment of gene expression from the IgG fraction using the 2^−ΔΔCt^ method [33]. To obtain enough ChIP-DNA for genomic microarray hybridization, IP and input DNA were amplified twice using the Sigma GenomePlex WGA2 kit (Sigma-Aldrich).

Biological replicates of WGA amplicons were first tested on hybrid arrays we designed to explore different regions of the human genome. These arrays consisted of CpG islands (20,000 probes), gene promoters (20,000 probes) and intergenomic regions (10,000 probes), allowing us to assess the binding distribution of each factor in these different regions (Custom array from Agilent, Santa Clara, CA, USA). Next, WGA amplicons were applied to Agilent Human Promoter ChIP on chip arrays 244K, containing 17,000 60-mer probes per array (Agilent Technologies, Santa Clara, CA, USA) also including miRNA gene promoters. The labeling of DNA samples for ChIP-chip analysis was performed using procedures previously described [34]. The arrays were scanned at 5-μm resolution on a scanner G2505C (Agilent Technologies) and signal intensities from the IP and the input fractions were compared after normalization. The data are accessible through GEO series access number GSE 106597.

### ChIP data analysis and computational motif discovery

Probes on ChIP-arrays were considered positive when signal intensities were significantly higher in the IP sample than in the control input sample. The selection of such positive probes was performed using an *ad hoc* statistical test adapted to non-tiling chips. Each probe was associated to a pair (x,y) of normalized log-transformed intensity, where x corresponded to the input and y to the IP sample. One important assumption was that points corresponding to non-positive probes were symmetrically distributed around the x=y axis. A threshold spline curve was then defined only considering the probes beyond this axis. Reporting this curve beyond the spline threshold allowed selecting positive probes. It also allowed defining p-values by using normalized distances between probes and the x=y axis. Considering replicates for each transcription factor, the final positive probes were selected using the two distinct p-values (pVal1 and pVal2) as follows:

$$\sqrt{pVal1*pVal2}<10e^{-3}\&pVal1<10e^{-2}\&pVal2<10e^{-2}$$.

The motif discovery task was performed on positive probes with flanking masked sequences of 200 bp. Then, the MD module in the TFBS tools (R package) allowed discovering motifs of 7 to 18 bases length [35]. In order to eliminate the redundancies between all the discovered motifs and to compare them with Jaspar and Transfac databases, the T-Reg Comparator tool was used [36]. Similar motifs were gathered leading to the definition of a *consensus* motif. Statistical significance for these motifs was computed regarding their score distribution in a set of positive and negative probes with a Chi-square homogeneity test [37]. Gene ontology enrichments for positive genes on ChIP-chip were determined using GOMiner.

### Quantitative RT-PCR analysis

Total RNA from 24h and 48h of 10% serum induced XTC.UC1 cells was isolated using the RNeasy kit (Qiagen). RNA integrity was determined using a Bio-Analyzer 2100 (Agilent Technologies). Reverse transcription was performed on 1μg of RNA with the Advantage RT-for-PCR kit (Clontech, Palo Alto, CA, USA) following the manufacturer’s recommendations. Real-time quantification was performed in a 96-well plate using the IQ SYBR Green Supermix and Chromo4 detector (BioRad, Hercules, CA, USA). Eleven genes were tested for quantitative expression: *CAMK2A, NDUFC1, POLG2, CYCS, UQCR, HIF1, CITED2, UCP2, COX18, COX4I2*. These corresponded to positive genes for both PRC and ERR ChIP-chip analyses after 48h of 20% serum induction. In all cases, mRNA expression data were normalised to β-globin. The sequences of primers used are listed in Supplementary Table 1.

## SUPPLEMENTARY MATERIALS FIGURES AND TABLES


