# Supplementary material for: Transcriptional orchestration of mitochondrial homeostasis in a cellular model of PGC-1-related coactivator-dependent thyroid tumor
Source: Oncotarget. 2018 Mar 23;9(22):15883–94. doi: 10.18632/oncotarget.24633 (PMC5882305; doi:10.18632/oncotarget.24633)
Supplement: Supplementary file 1 [file oncotarget-09-15883-s001.pdf]

Transcriptional orchestration of mitochondrial homeostasis in a cellular model of PGC-1-related coactivator-dependent thyroid tumor

SUPPLEMENTARY MATERIALS

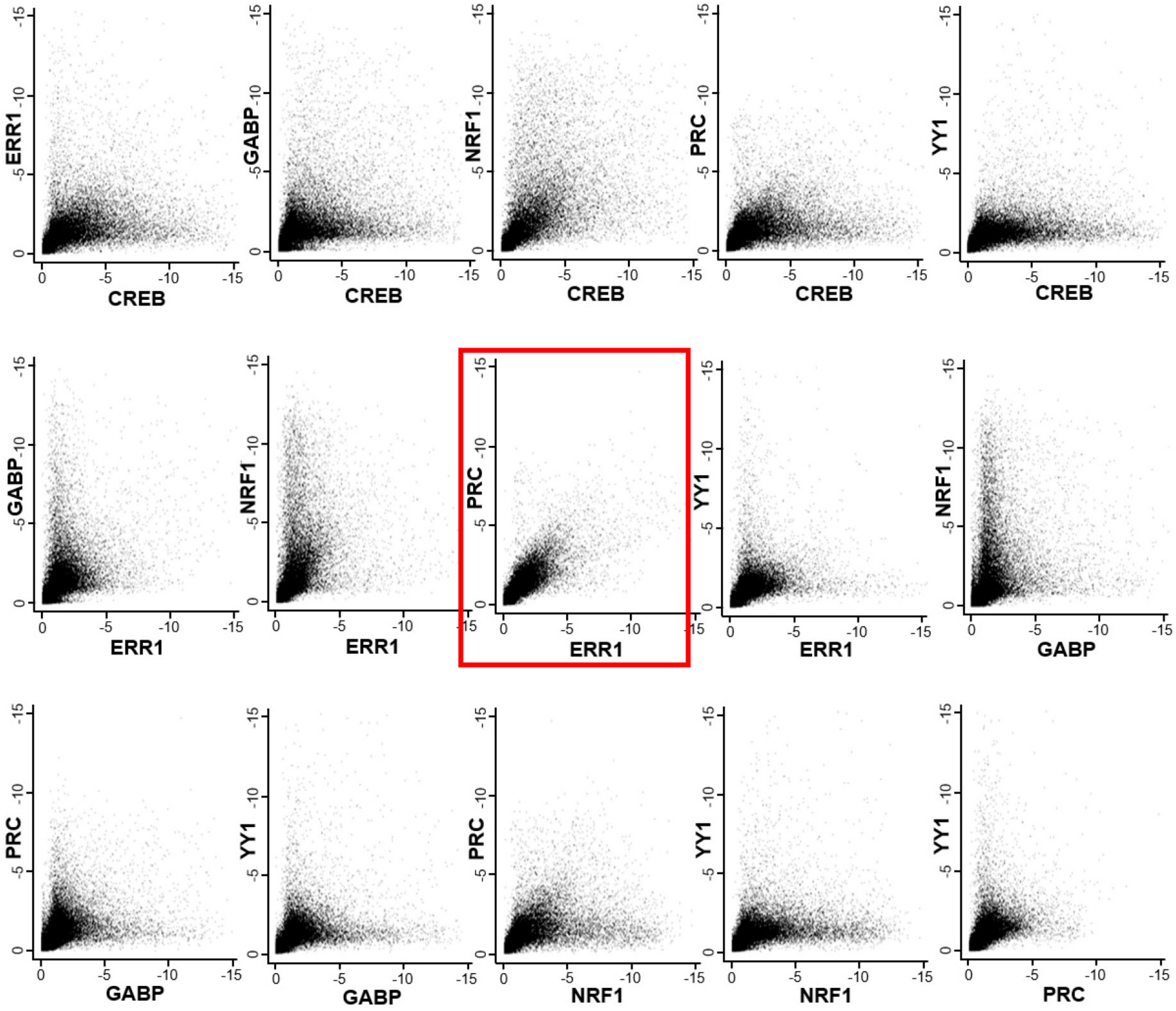

Supplementary Figure 1: Correlation between the best probes from 48h ChIP-chip analyses issued from two different factors. Each graph showed the correlation of the geometric mean (log base 10) for the best probe of each gene for two transcription factors. The red box corresponded to the correlation between ERR1 (ERRa) and PRC probes (Pearson correlation 0.82, p-value < 2.2 10^{-16}).

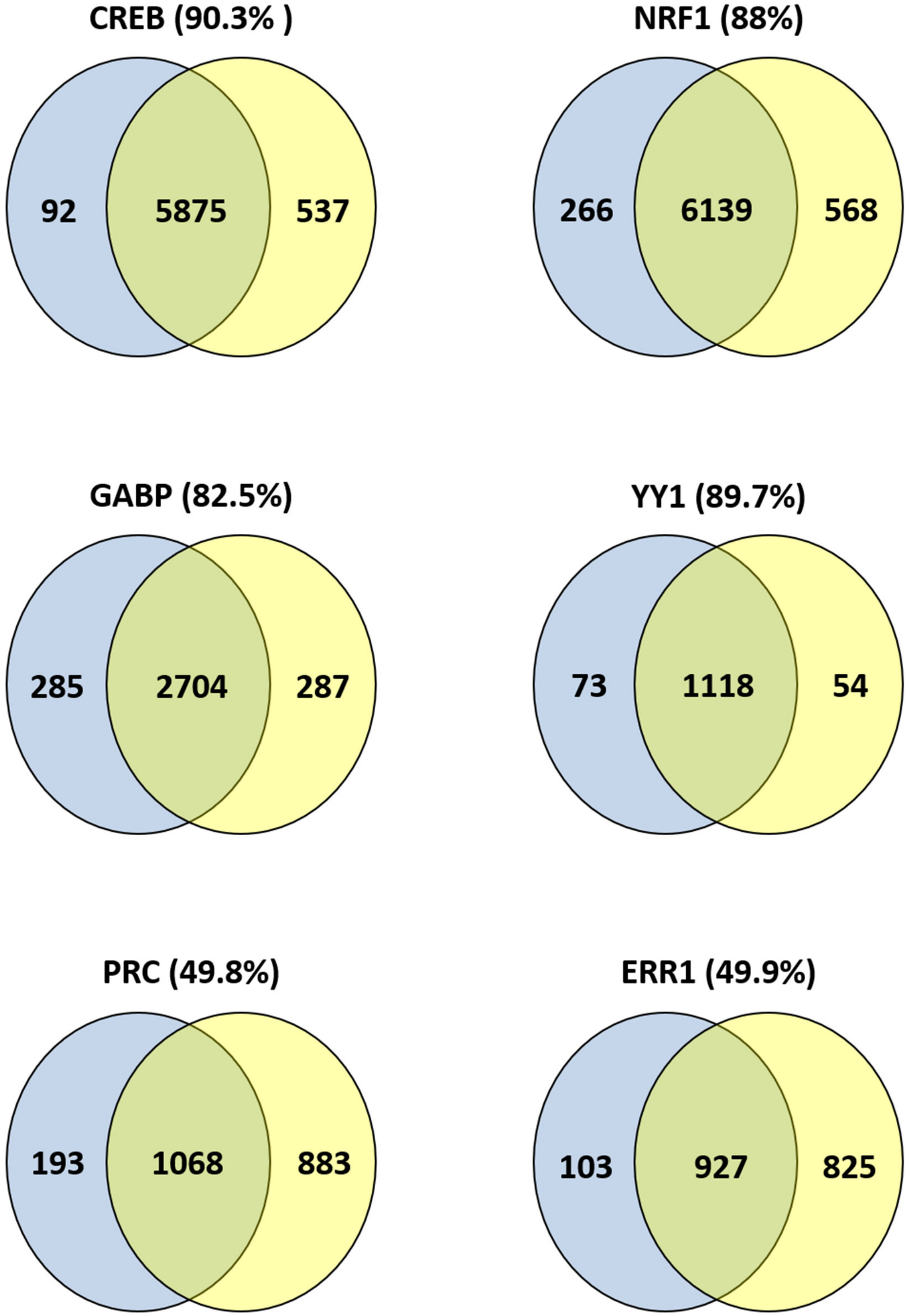

Supplementary Figure 2: Venn diagrams representing the number of positive genes on ChIP-chip analyses after 24h (blue) and 48h (yellow) of serum induction were shown for the six factors studied (CREB, NRF1, GABP, YY1, PRC and ERR1). The percentage of genes commonly positive at 24h and 48h are indicated in brackets.

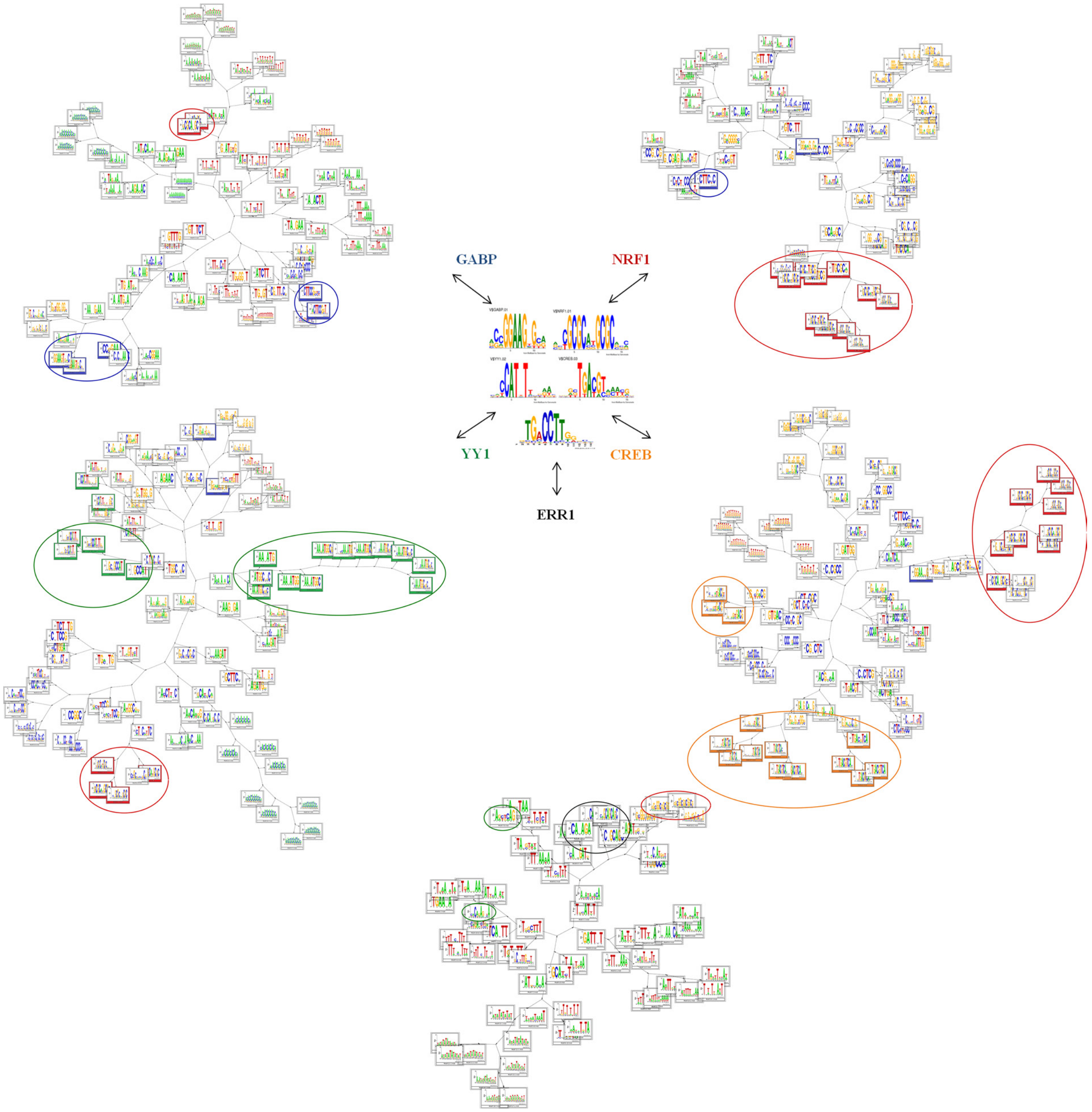

Supplementary Figure 3: Motif discovery in positive ChIP-chip promoters for NRF1, GABP, CREB, ERR1 and YY1. Colored circles represent the motifs discovered in gene promoters tested relative to *consensus* motif indicated in the center of the figure. Motifs discovered for other transcription factors than those explored by ChIP-chip are also shown by the corresponding color code. A high-resolution version of this figure is available on Oncotarget website.

PRC

GO.ID	Total	Change	Enrichissement	P.Value	Term
785	157	28	2.71	0	**chromatin**
786	55	14	3.87	0	nucleosome
1501	289	38	2.00	0	skeletal system development
1503	123	21	2.60	0	ossification
1568	240	36	2.28	0	blood vessel development
1666	79	16	3.08	0	**response to hypoxia**
1944	244	37	2.31	0	vasculature development
5694	388	47	1.84	0	chromosome
6333	113	21	2.83	0	**chromatin assembly or disassembly**
6334	69	15	3.31	0	nucleosome assembly
6793	1106	111	1.53	0	phosphorus metabolic process
6796	1106	111	1.53	0	phosphate metabolic process
6928	501	57	1.73	0	cell motion
8219	979	99	1.54	0	cell death
8283	945	100	1.61	0	**cell proliferation**
8285	305	40	1.99	0	**negative regulation of cell proliferation**
9653	1036	106	1.56	0	anatomical structure morphogenesis
9888	604	69	1.74	0	tissue development
9890	472	57	1.84	0	**negative regulation of biosynthetic process**
9892	631	76	1.83	0	**negative regulation of metabolic process**
9967	246	34	2.10	0	**positive regulation of signal transduction**
10558	454	55	1.84	0	**negative regulation of macromolecule biosynthetic process**
10605	595	69	1.76	0	**negative regulation of macromolecule metabolic process**
10627	217	32	2.24	0	regulation of protein kinase cascade
10740	150	24	2.43	0	**positive regulation of protein kinase cascade**
10926	922	109	1.80	0	anatomical structure formation
10941	657	70	1.62	0	regulation of cell death
16265	983	99	1.53	0	death
16477	317	43	2.06	0	cell migration
22607	668	76	1.73	0	cellular component assembly

ERRα

GO.ID	Total	Change	Enrichissement	P.Value	Term
785	157	42	2.15	0	chromatin
786	55	19	2.77	0	nucleosome
4725	92	27	2.35	0	**protein tyrosine phosphatase activity**
5925	93	26	2.24	0	focal adhesion
6333	113	33	2.34	0	chromatin assembly or disassembly
6357	607	116	1.53	0	**regulation of transcription from RNA polymerase II promoter**
8134	468	89	1.53	0	**transcription factor binding**
8138	40	18	3.61	0	**protein tyrosine/serine/threonine phosphatase activity**
9653	1036	198	1.53	0	anatomical structure morphogenesis
9790	434	83	1.53	0	embryonic development
9986	253	56	1.78	0	cell surface
10628	464	88	1.52	0	**positive regulation of gene expression**
10926	922	177	1.54	0	anatomical structure formation
16564	263	57	1.74	0	**transcription repressor activity**
17017	13	8	4.94	0	**MAP kinase tyrosine/serine/threonine phosphatase activity**
30055	100	28	2.25	0	cell-substrate junction
32989	359	71	1.59	0	cellular component morphogenesis
33549	13	8	4.94	0	**MAP kinase phosphatase activity**
43353	7	6	6.87	0	enucleate erythrocyte differentiation
44427	319	65	1.63	0	chromosomal part
45893	383	77	1.61	0	**positive regulation of transcription. DNA-dependent**
48646	310	67	1.73	0	anatomical structure formation involved in morphogenesis
51093	478	92	1.54	0	negative regulation of developmental process
51254	385	77	1.60	0	**positive regulation of RNA metabolic process**
65003	501	94	1.50	0	macromolecular complex assembly
902	328	65	1.59	1.00E-04	cell morphogenesis
5694	388	75	1.55	1.00E-04	chromosome
5924	97	26	2.15	1.00E-04	cell-substrate adherens junction
6605	217	47	1.74	1.00E-04	protein targeting
6869	138	30	1.74	0.0015	**lipid transport**

NRF1

GO.ID	Total	Change	Enrichissement	P.Value	Term
75	81	41	1.80	0	**cell cycle checkpoint**
226	127	61	1.71	0	microtubule cytoskeleton organization
278	422	178	1.50	0	**mitotic cell cycle**
775	102	48	1.68	0	chromosome. centromeric region
785	157	76	1.73	0	chromatin
4842	126	69	1.95	0	**ubiquitin-protein ligase activity**
5635	175	80	1.63	0	nuclear envelope
5643	68	35	1.84	0	nuclear pore
5694	388	180	1.65	0	chromosome
5759	208	95	1.63	0	**mitochondrial matrix**
5819	111	56	1.80	0	spindle
6091	301	129	1.53	0	**generation of precursor metabolites and energy**
6119	84	41	1.74	0	**oxidative phosphorylation**
6260	207	88	1.52	0	DNA replication
6325	344	149	1.54	0	chromatin organization
6333	113	58	1.83	0	chromatin assembly or disassembly
6396	505	219	1.55	0	RNA processing
6399	105	53	1.80	0	tRNA metabolic process
6412	370	158	1.52	0	translation
6457	154	71	1.64	0	protein folding
6511	212	93	1.56	0	**ubiquitin-dependent protein catabolic process**
6886	411	178	1.54	0	intracellular protein transport
6916	193	84	1.55	0	anti-apoptosis
7005	106	51	1.72	0	**mitochondrion organization**
7093	49	28	2.04	0	**mitotic cell cycle checkpoint**
8380	274	116	1.51	0	RNA splicing
10608	177	75	1.51	0	**posttranscriptional regulation of gene expression**
15630	456	193	1.51	0	microtubule cytoskeleton
16538	15	12	2.85	0	**cyclin-dependent protein kinase regulator activity**
16567	117	64	1.95	0	**protein ubiquitination**

GABP

GO.ID	Total	Change	Enrichissement	P.Value	Term
375	152	48	2.04	0	**RNA splicing. via transesterification reactions**
377	152	48	2.04	0	**RNA splicing. via transesterification reactions with bulged adenosine as nucleophile**
398	152	48	2.04	0	**nuclear mRNA splicing. via spliceosome**
3723	647	155	1.55	0	RNA binding
3735	148	51	2.23	0	structural constituent of ribosome
5654	753	188	1.61	0	nucleoplasm
5657	34	16	3.04	0	**replication fork**
5681	129	48	2.41	0	**spliceosomal complex**
5730	643	174	1.75	0	nucleolus
5740	355	85	1.55	0	**mitochondrial envelope**
5840	186	62	2.16	0	ribosome
6396	505	157	2.01	0	RNA processing
6397	301	95	2.04	0	mRNA processing
6399	105	41	2.53	0	tRNA metabolic process
6412	370	108	1.89	0	translation
6886	411	101	1.59	0	**intracellular protein transport**
8033	66	25	2.45	0	tRNA processing
8380	274	91	2.15	0	**RNA splicing**
16071	346	102	1.91	0	mRNA metabolic process
22613	165	56	2.19	0	**ribonucleoprotein complex biogenesis**
22904	52	20	2.49	0	**respiratory electron transport chain**
30529	447	147	2.13	0	**ribonucleoprotein complex**
31145	63	23	2.36	0	anaphase-promoting complex-dependent proteasomal ubiquitin-dependent protein catabolic process
31966	335	80	1.54	0	**mitochondrial membrane**
31974	1637	400	1.58	0	membrane-enclosed lumen
31981	1286	327	1.64	0	nuclear lumen
33279	118	39	2.14	0	**ribosomal subunit**
34470	164	54	2.13	0	**ncRNA processing**
34613	441	107	1.57	0	cellular protein localization
34660	204	71	2.25	0	ncRNA metabolic process

CREB

GO.ID	Total	Change	Enrichissement	P.Value	Term
75	81	45	1.74	0	**cell cycle checkpoint**
87	238	129	1.70	0	**M phase of mitotic cell cycle**
151	77	45	1.84	0	**ubiquitin ligase complex**
226	127	65	1.61	0	microtubule cytoskeleton organization
278	422	221	1.64	0	**mitotic cell cycle**
279	334	169	1.59	0	**M phase**
280	236	128	1.70	0	**nuclear division**
502	46	28	1.91	0	**proteasome complex**
775	102	65	2.00	0	**chromosome. centromeric region**
776	61	42	2.16	0	**kinetochore**
777	42	29	2.17	0	**condensed chromosome kinetochore**
779	46	30	2.05	0	**condensed chromosome. centromeric region**
785	157	85	1.70	0	**chromatin**
793	98	55	1.76	0	**condensed chromosome**
910	35	23	2.06	0	**cytokinesis**
3697	55	35	2.00	0	single-stranded DNA binding
3713	201	100	1.56	0	**transcription coactivator activity**
3723	647	312	1.51	0	RNA binding
3735	148	79	1.68	0	structural constituent of ribosome
4298	19	15	2.48	0	threonine-type endopeptidase activity
4386	125	69	1.73	0	helicase activity
4842	126	67	1.67	0	**ubiquitin-protein ligase activity**
5635	175	100	1.79	0	nuclear envelope
5643	68	45	2.08	0	nuclear pore
5654	753	360	1.50	0	nucleoplasm
5657	34	24	2.22	0	**replication fork**
5681	129	70	1.70	0	spliceosomal complex
5694	388	214	1.73	0	**chromosome**
5759	208	100	1.51	0	mitochondrial matrix
6281	263	133	1.59	0	**DNA repair**

YY1

GO.ID	Total	Change	Enrichissement	P.Value	Term
313	49	14	5.40	0	**organellar ribosome**
314	19	7	6.96	0	**organellar small ribosomal subunit**
375	152	27	3.35	0	RNA splicing. via transesterification reactions
377	152	27	3.35	0	RNA splicing. via transesterification reactions with bulged adenosine as nucleophile
398	152	27	3.35	0	nuclear mRNA splicing. via spliceosome
3723	647	83	2.42	0	RNA binding
3735	148	29	3.70	0	**structural constituent of ribosome**
5739	966	94	1.84	0	**mitochondrion**
5759	208	31	2.81	0	**mitochondrial matrix**
5761	49	14	5.40	0	**mitochondrial ribosome**
5763	19	7	6.96	0	**mitochondrial small ribosomal subunit**
5840	186	34	3.45	0	**ribosome**
6396	505	72	2.69	0	RNA processing
6397	301	45	2.82	0	mRNA processing
6412	370	61	3.11	0	**translation**
8380	274	42	2.89	0	RNA splicing
10467	3247	280	1.63	0	gene expression
16070	2157	180	1.58	0	**RNA metabolic process**
16071	346	48	2.62	0	**mRNA metabolic process**
16607	93	19	3.86	0	nuclear speck
30529	447	61	2.58	0	ribonucleoprotein complex
31974	1637	136	1.57	0	membrane-enclosed lumen
31980	208	31	2.81	0	**mitochondrial lumen**
32991	2342	194	1.56	0	macromolecular complex
33279	118	20	3.20	0	**ribosomal subunit**
34660	204	28	2.59	0	**ncRNA metabolic process**
43233	1607	135	1.59	0	organelle lumen
44267	2354	193	1.55	0	cellular protein metabolic process
44429	518	58	2.11	0	**mitochondrial part**
42773	45	10	4.20	1.00E-04	**ATP synthesis coupled electron transport**

Supplementary Figure 4: For each transcription factor and PRC coactivator most representative gene ontologies (n=30) for positive genes on ChIP-chip were determined using GOMiner software.

Supplementary Table 1: Primers used for quantitative RT-PCR analysis

CYCS	For: 5′-CCAGTGCCACACCGTTGAA-3′ Rev: 5′-TCCCCAGATGATGCCTTTGTT-3′;
CITED2	For: 5′-GCGAAGGAGGATGCCAACC-3’ Rev: 5′-CAGCTGCATACTAGCCAGCA-3′
COX18	For: 5’-CATCAGCACCACTTCACGAA-3’ Rev: 5’-CCACCAACTGTCAGAAGCAT-3’
CAMK2A	For: 5’-TACGGCGACGAGAAGCTGAG-3’ Rev: 5’-AGACACCAGGAGGTGCCTTG-3’
COX4I2	For: 5’-GGTGGAAGACGAGGGATGCA-3’ Rev: 5’-CAGCTGGGTCCAGCTTCCCT-3’
HIF1	For: 5’-AGGAGCTGAGTTGGAATAGTG-3’ Rev: 5’-CCAGCTATCTTACTAGTGGGTG-3’
NDUFC1	For: 5’-GCCGTCTGGTTACTGTAATC-3’ Rev: 5’-GGTTACTCAGTGTCATCTTG-3’
POLG2	For: 5’-CAACGGAGGTGAGCGTGC-3’ Rev: 5’-GAGGTGTATATAGAAGGTGAT-3’
UCP2	For: 5’-CATTCTGACCATGGTGCGTACTGA-3’ Rev: 5’-GTTCATGTATCTCGTCTTGACCAC-3’
UQCR	For: 5’-CTTCCTGGGCCCACGCTACC-3’ Rev: 5’-GTCCAGGATCAGCCGCCAAT-3’
β-globin	For: 5′-CAACTTCATCCACGTTCACC-3′ Rev: 5′-ACACAACTGTGTTCACTAG-3’
